# Comparison of three PET methods to assess peritoneal membrane transport

**DOI:** 10.1590/1414-431X20198596

**Published:** 2019-08-05

**Authors:** RF. Romani, J. Waniewski, L. Kruger, B. Lindholm, M.M. Nascimento

**Affiliations:** 1 Departamento de Nefrologia, Hospital Universitário Evangélico de Curitiba, Curitiba, PR, Brasil; 2 Nalecz Institute of Biocybernetics and Biomedical Engineering, Polish Academy of Sciences, Warsaw, Poland; 3 Renal Medicine and Baxter Novum, Department of Clinical Sciences and Intervention, Karolinska Institute, Stockholm, Sweden

**Keywords:** Peritoneal dialysis, Peritoneal membrane, Peritoneal equilibration test, Dialysis adequacy, Peritoneal solute transport

## Abstract

The peritoneal equilibration test (PET) is the most widespread method for assessing water and solute transport across the peritoneal membrane. This study compared three methods: traditional PET (t-PET), mini-PET, and modified PET (mod-PET). Non-diabetic adults (n=21) who had been on peritoneal dialysis (PD) for at least three months underwent t-PET (glucose 2.5%-4 h), mini-PET (glucose 3.86%-1 h), and mod-PET (glucose 3.86%-4 h) to determine dialysate-to-plasma concentration ratio (D/P) for creatinine and dialysate-to-baseline dialysate concentration ratio (D/D0) for glucose. Agreement between methods regarding D/P creatinine and D/D0 glucose was assessed using analysis of variance (ANOVA), Pearson's correlation coefficient, and Bland-Altman analysis. D/P creatinine differed between t-PET and mini-PET (P<0.001) and between mod-PET and mini-PET (P<0.01) but not between t-PET and mod-PET (P=0.746). The correlation of D/P creatinine with t-PET *vs* mod-PET was significant (r=0.387, P=0.009) but not that of t-PET *vs* mini-PET (r=0.088, P=0.241). Estimated bias was −0.029 (P=0.201) between t-PET and mod-PET, and 0.206 (P<0.001) between t-PET and mini-PET. D/D0 glucose differed between t-PET and mod-PET (P=0.003) and between mod-PET and mini-PET (P=0.002) but not between t-PET and mini-PET (P=0.885). The correlations of D/D0 glucose in t-PET *vs* mod-PET (r=−0.017, P=0.421) or t-PET *vs* mini-PET (r=0.152, P=0.609) were not significant. Estimated bias was 0.122 (P=0.026) between t-PET and mod-PET, and 0.122 (P=0.026) between t-PET and mini-PET. The significant correlation of D/P creatinine between t-PET and mod-PET suggested that the latter is a good alternative to t-PET. There was no such correlation between t-PET and mini-PET.

## Introduction

Peritoneal dialysis (PD) is a modality of renal replacement therapy in which the peritoneal membrane (PM), semipermeable to water and solutes, allows the diffusion of solutes between the uremic plasma and the dialysate residing in the peritoneal cavity (1). During the PD process, the transport characteristics of the PM differ between patients with regard to the rate of transport of solutes and water and this may have clinical implications on dialysis therapy prescription (2). Therefore, to achieve the best possible treatment, patients need to be classified according to the PM transport profile (3,4).

For this purpose, the peritoneal equilibrium test (known by its acronym PET) was described in 1987 by Twardowski et al. (5) who classified the PM solute transport profile of a patient according to the membrane's permeability to two small molecules, creatinine, MW 113, and glucose, MW 180, using dialysis fluid with 2.5/2.27% anhydrous glucose as osmotic agent. An alternative to the traditional PET (t-PET) is a similar test that uses a solution with a higher concentration (4.25/3.86% of glucose), known as hypertonic PET or modified PET (mod-PET). The mod-PET is advocated by the International Society for Peritoneal Dialysis (6). The results obtained with mod-PET for solute transport appear to be comparable to those of t-PET, but mod-PET provides additional information such as the indirect assessment of free water transport across the peritoneal water-only channels (aquaporins) through the determination of sodium transport and sieving of sodium.

Another alternative test is the mini-PET, which was introduced in 2005 by La Milia et al. (7). It uses bags with 2 L of fluid with 4.25/3.86% glucose, but the dialysate is drained after only 1 h. It appears to provide information equivalent to the t-PET with regard to the transport of small molecules (7).

The aim of the present study was to compare these three methods (mini-PET, mod-PET, and t-PET) in the assessment of PM small-solute transport profile in patients with chronic kidney disease (CKD) undergoing PD.

## Material and Methods

This study was part of a collaboration between the Fundação Pró-Renal (FPR), Brazil, Karolinska Institute, Sweden, and Polish Academy of Sciences, Poland, in evaluating the transport of solutes and insulin resistance in patients undergoing PD. One hundred and eighty-eight patients on PD from the FPR, aged ≥18 years, were included in the initial evaluation. Patients with diabetes and patients who had been on PD for less than three months were excluded. Twenty-one patients who agreed to participate were selected through convenience sampling.

The PM transport profile of these patients was assessed using t-PET and mini-PET, and a 4-hour dialysis session with dialysis fluid containing 3.86% glucose was subsequently performed with the collection of serial blood and dialysate samples (mod-PET). Commercially available dialysis solutions (Dianeal^®^, Baxter Healthcare Corporation, USA) in bags for manual infusion were used. The composition of the solution was as follows: 132 mEq/L sodium, 95 mEq/L chloride, 2.5 mEq/L calcium, 0.5 mEq/L magnesium, and 40 mEq/L lactate (Dianeal^®^, Baxter Healthcare Corporation).

Written informed consent was obtained from all patients and the study protocol was approved by the Ethics Committee of the Faculdade Evangélica do Paraná (FEPAR), under protocol number 7876/11 (12 June 2011).

### t-PET

All tests were performed in the Dialysis Center by a trained nursing team and under the supervision of a medical team. The standard procedure began with the complete drainage of remaining fluid from the patient's peritoneal cavity, and the 2.27% glucose dialysis solution was then infused. The dwell time was 4 h. Dialysate samples (10 mL) were collected at 0 min (immediately after infusion), and 2 and 4 h after infusion. A blood sample (10 mL) was collected at 2 h. The infusion initiation and drainage times were recorded (8).

### mini-PET

The standard procedure began with drainage of fluid from the peritoneal cavity and then the 3.86% glucose dialysis solution was infused. The dwell time was 1 h. The dialysate samples (10 mL) were collected at 0 h (immediately after infusion) and at 1 h after infusion. A blood sample (10 mL) was collected at 1 h. The infusion initiation and drainage times were recorded. These tests were performed at least 48 h after performance of the t-PET (8).

### mod-PET

The standard procedure began with drainage of fluid from the patient's peritoneal cavity followed by the infusion of the 3.86% glucose dialysis solution with a dwell time of 4 h. Dialysate samples (10 mL) were collected every hour, at 0 h (immediately after infusion), and 1, 2, 3, and 4 h after infusion. Blood samples (10 mL) were collected every 30 min, at 0 h (immediately after infusion), and at 0.5, 1, 1.5, 2, 2.5, 3, 3.5, and 4 h after infusion. Dialysis infusion initiation and drainage times were recorded. The test was performed at least 48 h after the application of the mini-PET. The dialysate samples collected at 0, 2, and 4 h and the blood samples collected at 2 h were used to calculate the results of mod-PET (8).

### Analyses

The blood and dialysate samples were obtained between April 2012 and January 2013 according to the protocols described above and were kept in cryogenic storage (–30°C) until they were sent for analysis at the Karolinska Institute in Stockholm, Sweden.

For the purposes of the study, the concentrations of creatinine and glucose in blood and dialysate samples were determined. Glucose levels (mmol/L) were measured using the colorimetric enzymatic method, and creatinine levels (umol/L) were determined by the Jaffé method. The blood sample of one patient used in the mod-PET could not be analyzed due to hemolysis and was excluded from the final analysis.

The determination of creatinine levels in the dialysate using the Jaffé method is affected by the high glucose concentration. The falsely high results produced by this method must be corrected and a correction factor must be provided by the laboratory (9). The correction factor applied in this protocol was: creatinine level –0.234 × glucose level

The creatinine result of one patient in the mod-PET, glucose results of two patients in the mini-PET, and the glucose result of one patient in the mod-PET were excluded because of sample analysis problems.

The dialysate-to-plasma concentration ratio (D/P) for creatinine and the dialysate-to-baseline dialysate concentration ratio (D/D0) for glucose were calculated for the three tests using Microsoft Excel^®^ software, version 14.00 for Mac (Microsoft Corporation, USA).

#### D/P creatinine

a) D/P creatinine in the t-PET was calculated using the results of the dialysate samples at 0, 2, and 4 h in the numerator and the results of the plasma samples in the denominator. The value obtained at 4 h was used as reference for analysis (5,10); b) D/P creatinine in the mini-PET was calculated using the results of creatinine in the dialysate samples at 0 and 1 h in the numerator and the results of the plasma samples in the denominator (10); c) D/P creatinine in the mod-PET was calculated using the creatinine results of the dialysate samples at 0, 2, and 4 h in the numerator and the results of the plasma samples in the denominator. The value obtained at 4 h was used as reference for analysis (10).

#### D/D0 glucose

a) D/D0 glucose in the t-PET was calculated using the glucose results of the dialysate samples at 4 h in the numerator and at 0 h in the denominator (10); b) D/D0 glucose in the mini-PET was calculated using the glucose results of the dialysate samples at 1 h in the numerator and at 0 h in the denominator (10); c) D/D0 glucose in the mod-PET was calculated using the glucose results of the dialysate samples at 4 h in the numerator and at 0 h in the denominator (10).

### Statistical analysis

The results of quantitative variables are described as means, medians, minimum and maximum values, and standard deviations, as appropriate. Qualitative variables are described as frequencies and percentages. The repeated measures analysis of variance (ANOVA) was used to compare quantitative variables between the three methods (t-PET, mini-PET, and mod-PET). Multiple comparisons were performed using the test of least significant difference (LSD). The variables that did not meet the condition of normality (evaluated using the Kolmogorov-Smirnov test) were subject to logarithmic transformation. To assess the inter-method agreement regarding transport classification (low, average, or high), the Kappa coefficient of agreement was estimated. The correlation between the methods was determined using Pearson's coefficient of correlation, and Bland-Altman analysis was used to assess the agreement between the techniques. Values of P<0.05 indicated statistical significance. The data were analyzed using the SPSS Statistics for Windows, version 20.0. (USA).

## Results

Clinical and demographic data of study participants (n=21) are summarized in Table 1.

**Table 1.** Clinical and demographic characteristics of 21 peritoneal dialysis (PD) patients.

Gender (M), n (%)	15 (71)
Age (years)	52±15
Time on PD (months)	18±14
Underlying disease	SAH: 6; Glomerulopathy: 10; Other: 5
Smoking (%)	14
Dyslipidemia (%)	28
BMI (kg/m^2^)	23.4±3.05
Hemoglobin (g/dL)	12.2±1.9
Serum calcium (mg/dL)	9.02±0.59
Phosphorous (mg/dL)	4.53±1.73
iPTH (ng/mL)	357±276
Urea (mg/dL)	113±29
Serum albumin (g/dL)	3.8±0.69

Data are reported as means±SD. SAH: systemic arterial hypertenion; BMI: body mass index; iPTH: intact parathyroid hormone.

### D/P Creatinine

**Figure 1.**
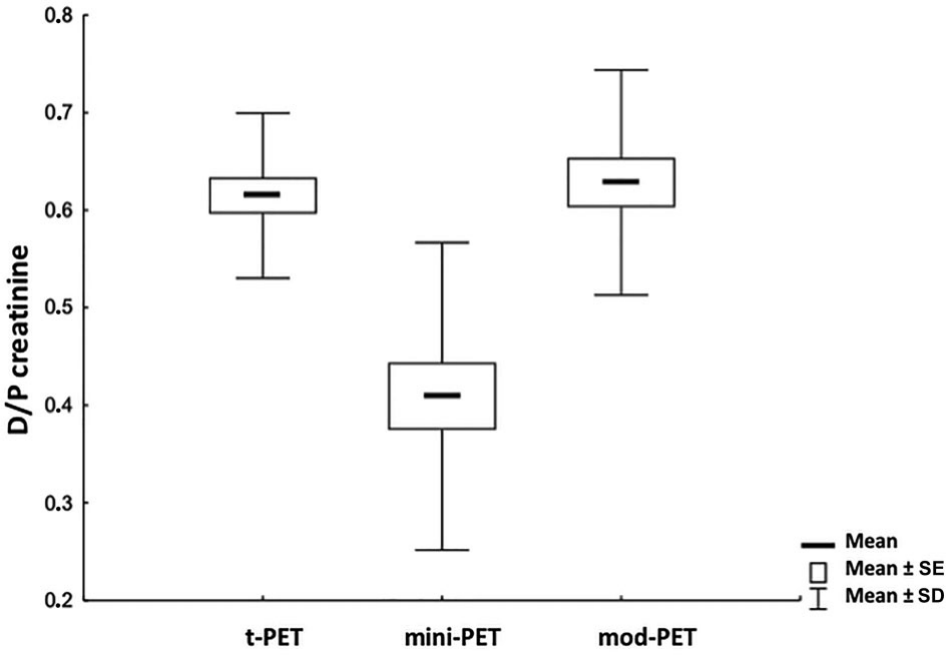
Dialysate-to-plasma concentration ratio (D/P) for creatinine in traditional peritoneal equilibration test (t-PET) *vs* mini-PET *vs* modified (mod)-PET (n=20). According to the repeated measures ANOVA, D/P creatinine did not differ between t-PET and mod-PET (P=0.746), but there was a significant difference between t-PET and mini-PET (P<0.001) and between mod-PET and mini-PET (P>0.001).

Repeated measures ANOVA did not show a significant difference in D/P creatinine between t-PET and mod-PET (P=0.746), but there was a significant difference between t-PET and mini-PET (P<0.001) and between mod-PET and mini-PET (P>0.001, Figure 1).

**Figure 2.**
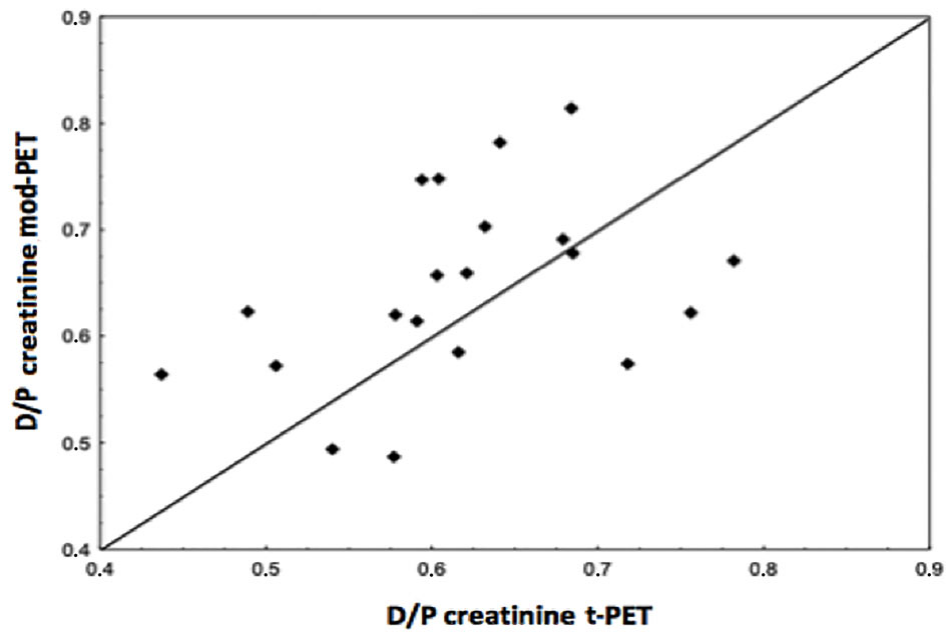
Correlation of dialysate-to-plasma concentration ratio (D/P) for creatinine between traditional peritoneal equilibration test (t-PET) *vs* modified (mod)-PET (r=0.387, P=0.009; n=20).

In addition, there was a significant positive correlation between t-PET and mod-PET (r=0.387, P=0.009, Figure 2) but not between t-PET and mini-PET (r=0.088, P=0.241).

**Figure 3.**
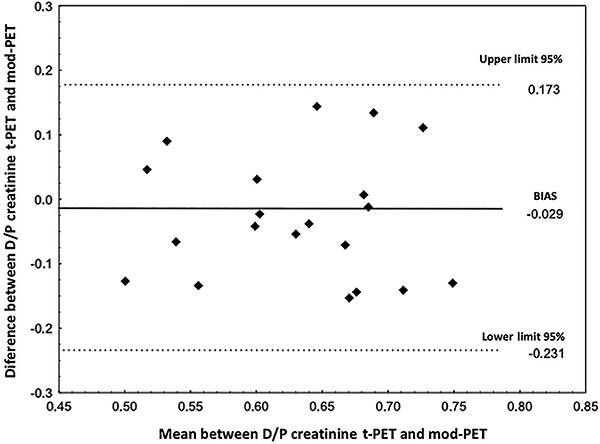
Bland-Altman analysis for dialysate-to-plasma concentration ratio (D/P) for creatinine between traditional peritoneal equilibration test (t-PET) *vs* modified (mod)-PET (n=20). There was no significant bias between t-PET and mod-PET (−0.029; P=0.201).

The bias between t-PET and mod-PET estimated using the Bland-Altman analysis was –0.029 (P=0.201), which indicated that the hypothesis of the absence of a bias between the results of PET and mod-PET was not rejected (Figure 3). The bias between t-PET and mini-PET was 0.206 (P<0.001), which indicated that the hypothesis of the absence of a bias between PET and mini-PET results was rejected.

Analysis of agreement between the methods with regard to membrane profile classification showed that there was no significant agreement between t-PET and mini-PET. The estimated Kappa coefficient of agreement was 0.01 (poor agreement) and not statistically significant (P=0.415). Moreover, there was no significant agreement between t-PET and mod-PET. The estimated Kappa coefficient of agreement was 0.08 (poor agreement), without statistical significance (P=0.676). The estimated Kappa coefficient of agreement between mini-PET and mod-PET was 0.07 (poor agreement; P=0.250).

The accuracy of the mod-PET in identifying high and low transporters was 65% in relation to t-PET.

### D/D0 Glucose

Repeated measures ANOVA showed significant differences in D/D0 glucose between t-PET and mini-PET (P=0.001), between t-PET and mod-PET (P=0.031), and between mod-PET and mini-PET (P<0.001).

In addition, no significant correlation was observed between t-PET and mod-PET (r=−0.017, P=0.421) and between t-PET and mini-PET (r=0.152, P=0.609).

The bias between t-PET and mod-PET estimated by Bland-Altman analysis was 0.122 (P=0.026), thus indicating that the hypothesis of the absence of a bias between the t-PET and mod-PET results was rejected. The estimated bias between t-PET and mini-PET was 0.122 (P=0.026), which indicated that the hypothesis of the absence of a bias between the PET and mini-PET results was rejected.

Analysis of agreement between the methods with regard to membrane profile classification showed that there was no significant agreement between t-PET and mini-PET. The estimated Kappa coefficient of agreement was −0.03 (poor agreement), without significance (P=0.627). Furthermore, there was no significant agreement between t-PET and mod-PET. The estimated Kappa coefficient of agreement was −0.09 (poor agreement), without statistical significance (P=0.494). The estimated Kappa coefficient of agreement between mini-PET and mod-PET was 0.01 (poor agreement), without statistical significance (P=0.847).

### Ultrafiltration

Analysis of variance showed a statistical difference between t-PET and mod-PET (P<0.001) with regard to ultrafiltration (UF); however, there was no statistical difference between t-PET and mini-PET (P=0.375).

There was no correlation between t-PET and mod-PET (r=−0.159, P=0.425); however, there was a correlation between t-PET and mini-PET (r=0.260, P=0.009).

The bias between t-PET and mod-PET estimated by Bland-Altman analysis was −473.3 (P<0.001), which indicated that the hypothesis of the absence of a bias between PET and mod-PET was rejected. The estimated bias between t-PET and mini-PET was −76.7 (P=0.426), thus indicating that the hypothesis of the absence of a bias between the t-PET and mini-PET results was not rejected.

## Discussion

### t-PET *vs* mod-PET

The results of the present study are in line with those obtained by Smit et al. (6) who compared peritoneal transport with 1.36 and 3.86% glucose solutions and did not find an effect of solute tonicity on the creatinine D/P ratio. Peritoneal permeability analysis performed in the same study demonstrated that there was no effect of dialysis solution tonicity on the area of small-solute mass transfer, which implies that there is no influence on effective peritoneal area. There was no correlation between the two methods with regard to D/D0 glucose because it was lower in the mod-PET. The authors suggested that this may be due to higher UF and consequent dilution of the dialysate (6). The results led the authors to conclude that the mod-PET is the test of choice because it provides information similar to that provided by the t-PET plus additional information on free-water transport and UF.

Pride et al. (11) compared t-PET and mod-PET with regard to the transport of creatinine, glucose, and sodium and, similar to the present study, obtained reproducibility in the classification of the transport across the membrane using D/P creatinine. The volume of UF and the evaluation of aquaporin function differed between the two methods. The authors concluded that the mod-PET provides additional information without prejudice to molecule transport classification.

La Milia et al. (12) performed a prospective analysis of 95 patients with annual application of the mod-PET for D/P creatinine, D/D0 glucose, UF, and sodium sieving. These authors observed a gradual drop in UF from the second year onwards. In multivariate analysis, the loss of aquaporin function, which was assessed by sodium sieving, correlated with the risk of UF loss (risk ratio (RR) 0.797 (0.649–0.965), P=0.020). They concluded that the mod-PET provides more information than t-PET, without prejudice to solute transport assessment, and that there is a gradual loss in aquaporin transport over the years on PD that correlates with UF failure.

The equivalence between t-PET and mod-PET with regard to transport profile assessment appears to be confirmed by our analysis. The data allowed inference that with a similar time of exposure to the dialysis solution, as occurs in the two tests, the effect of tonicity and subsequent solute (creatinine) convection have little effect on the result of the assessment of peritoneal transport by both methods.

The comparison between t-PET and mod-PET with regard to D/D0 glucose showed a significant difference; there was a bias between the methods and there was no correlation between linear values or the categorical classification. This difference may be explained by the different concentrations of glucose, and is similar to that obtained in other studies that compared tests with distinct glucose concentrations (6,11,12). D/P creatinine is, however, the most commonly used parameter in clinical decision-making and appears to be the best parameter for peritoneal transport classification tests with hypertonic glucose.

### t-PET *vs* mini-PET

Performing t-PET is laborious and time-consuming and, therefore, the use of simpler and quicker methods, such as the mini-PET, has been studied, with different results (13–18). La Milia et al. (7) demonstrated equivalence between mini-PET and t-PET in the characterization of small molecule transport. In addition, the study suggested the possibility of assessing free-water transport through the aquaporins.

Baştuğ et al. (17) demonstrated, using Pearson's correlation and bias assessment with the Bland-Altman test, that the characterization of transport using PET and mini-PET was similar, as was the categorical classification of high, average, and low transport using D/P creatinine evaluation. Kazancioğlu et al. (18) and Cano et al. (19) also demonstrated a positive correlation between t-PET and mini-PET, performed by infusing the dialysate for 2 h.

Cuevas et al. (16) conducted a prospective study of the results of 81 PET tests in 81 patients. They analyzed the results collected after 4 h (t-PET) and after 2 h (mini-PET) for glucose and creatinine and did not find a correlation between the results.

The comparison between t-PET and mini-PET in the present study with regard to D/P creatinine showed a significant difference; there was a bias between the methods and there was no correlation between the linear values or the categorical classification. Our results suggest that mini-PET was not equivalent to t-PET with regard to the classification of peritoneal transport, despite its ease of application.

### UF Assessment

UF failure is defined as UF lower than 400 mL after 4 h of exposure to a 3.36% glucose solution (20–24). According to Ho-dac-Pannekeet et al. (24), the use of a 3.36% glucose solution is better for UF evaluation as it reveals the true UF capacity of the PM because the proportion of UF flow through aquaporins is higher.

In the present study, a secondary analysis was performed of the correlation of UF of t-PET and mini-PET, respectively, with mod-PET, which is considered the reference test to evaluate UF failure. UF in the t-PET and mini-PET differed from that in the mod-PET and, according to our findings, these tests (i.e., t-PET and mini-PET) were not adequate for UF failure assessment.

The present study has some limitations. This was a small convenience sample and, therefore, the results should be carefully interpreted. The prevalence of high and average-high transporters was high, which may create a bias in the analysis of transport compared to other patient populations undergoing PD.

## Conclusion

This study confirmed that the rate of creatinine removal as assessed by D/P creatinine was similar with mod-PET and t-PET. This strengthened the evidence that mod-PET is a method that provides additional information (evaluation of UF and of free water transport) without prejudice to the evaluation of small solute transport as compared to t-PET. While the main advantages of the mini-PET are its fast execution and the possibility of assessing aquaporin function, its lack of equivalence to t-PET in the evaluation of solute removal as observed in the current study is a limitation. On the other hand, the majority of previous studies in adults or children show good correlation between evaluations of solute transport at 1, 2, or 4 h (7,15,17,19). We concluded that t-PET remained a reliable test for the study of solute transport across the PM; however, mod-PET has been shown to be a useful alternative when more detailed information about UF and free water transport are required.
